# What Does Eye-Blink Rate Variability Dynamics Tell Us About Cognitive Performance?

**DOI:** 10.3389/fnhum.2017.00620

**Published:** 2017-12-19

**Authors:** Rafal Paprocki, Artem Lenskiy

**Affiliations:** Korea University of Technology and Education, Cheonan, South Korea

**Keywords:** eye-blink rate variability dynamics, cognitive performance, dynamics of inter-blink intervals, blink-rate variability

## Abstract

Cognitive performance is defined as the ability to utilize knowledge, attention, memory, and working memory. In this study, we briefly discuss various markers that have been proposed to predict cognitive performance. Next, we develop a novel approach to characterize cognitive performance by analyzing eye-blink rate variability dynamics. Our findings are based on a sample of 24 subjects. The subjects were given a 5-min resting period prior to a 10-min IQ test. During both stages, eye blinks were recorded from Fp1 and Fp2 electrodes. We found that scale exponents estimated for blink rate variability during rest were correlated with subjects' performance on the subsequent IQ test. This surprising phenomenon could be explained by the person to person variation in concentrations of dopamine in PFC and accumulation of GABA in the visual cortex, as both neurotransmitters play a key role in cognitive processes and affect blinking. This study demonstrates the possibility that blink rate variability dynamics at rest carry information about cognitive performance and can be employed in the assessment of cognitive abilities without taking a test.

## 1. Introduction

A search^1^ request for the keyword “IQ” at http://pubmed.gov currently returns 19,599 results, with the number of articles growing every year. Obviously, the study of intelligence has been and continues to be a hot topic of research. Since Alfred Binet developed the first practical Intelligence Quotient (IQ) test (Becker, 2003), many forms of intelligence have been distinguished. Neisser et al. (1996) defined intelligence as the “ability to understand complex ideas, to adapt effectively to the environment, to learn from experience, to engage in various forms of reasoning, [and] to overcome obstacles by taking thought.” Later, Gardner (1999) gave a broad definition of intelligence, as being a “biopsychological potential to process information that can be activated in a cultural setting to solve problems or create products that are of value in a culture.” Clearly, this definition encapsulates various ways of defining intelligence.

A different approach to defining intelligence is based on the concept of multiple intelligences (MI). According to MI, there are eight types of intelligence: linguistic, logical-mathematical, visual-spatial, bodily-kinesthetic, musical, interpersonal, intrapersonal, and naturalistic. However, there is an ongoing debate whether the concept of MI has adequate experimental support and a neurophysiological foundation (Shearer and Karanian, 2017). Yet, another way of looking at intelligence is to consider it as either a single general concept or two distinctive concepts, called fluid and crystallized intelligence.

The problem of performance evaluation emerges regardless of the definition of intelligence. An IQ score is thought to be a measure of a person's performance and can be interpreted differently, depending on one's perspective. Some IQ tests only contain problems to assess fluid intelligence; such problems include mathematical analogies and logical and spatial problems. However, other IQ tests contain problems that require crystallized intelligence, i.e., problems that require prior knowledge or verbal problems. In our experiment, we selected only visual, spatial, and logical problems and removed all verbal problems from the IQ test we used, to focus solely on fluid intelligence or, in terms of the concept of MI, on visual-spatial and logical-mathematical intelligence.

The use of an IQ test allowed us to assess the subjects' cognitive performance by activating logical reasoning, visual imagination, and pattern-recognition skills. The work the subject performed to solve the test included the individual's perception of the amount and difficulty of the task, known as mental workload.

The research literature on physiological markers of mental workload includes an extensive body of research to characterize the type and intensity of cognitive processes. The processes related to mental workload are, for example, attention (Parasuraman, 1979), perception, memory, learning (Berka et al., 2007), language, and higher reasoning. It has been found that mental workload can be assessed by measuring heart and respiration rates, blood pressure, the skin potential response, blink rate (Takahashi et al., 1994) and dilation of the pupils.

Specifically, this research has documented that the pupils dilate momentarily under a mental load (Hess and Polt, 1964); in particular, during memory tasks (Beatty and Kahneman, 1966). It has been shown that pupil size correlates with intelligence while, on the other hand, a connection between pupil size and dopamine has been demonstrated (Spiers and Calne, 1969), indicating a connection between intelligence and dopamine level (Previc, 1999; Seamans and Robbins, 2010). It also has been demonstrated that blink patterns are related to certain types of mental workload (Bentivoglio et al., 1997). This research dates back to the work of Ponder and Kennedy (1927), who noticed that the rate of blinking is closely related to “mental tension.”

Eye-blink activity has been studied as an index of creativity, in relation to dopamine (Chermahini and Hommel, 2010), and emotional changes (Akbari Chermahini and Hommel, 2012). It is known that attentional control, which is a process ensuring that one's actions correspond with one's goals, is related to the magnitude of eye blinks (Peers et al., 2013). Blink rate (BR: the number of blinks per minute) during choice-response tasks can provide a reliable measure of cognitive processing (e.g., Wascher et al., 2015 in the central nervous system Ichikawa and Ohira, 2004). In particular, it has been shown that the endogenous eye blink is a response controlled by the cortex (Orchard and Stern, 1991). Its characteristics, like rate and temporal distribution, allow it to be distinguished from voluntary or reflexive eye-lid movements, and it seems to reflect cognitive states. Eye blinks indicate the reallocation of mental resources (e.g., while driving Benedetto et al., 2011), cognitive states (e.g., relaxed or engaged in problem solving Marshall, 2007, or transition points in the processing of information Martins and Carvalho, 2015). Although the rate of spontaneous (i.e., endogenous) eye blinks has been repeatedly found to be related to cognitive processes, it has been recently reported to be modified by level of attention while watching a television screen (Andreu-Sánchez et al., 2017).

There is ongoing research that relates eye-blink rate variability (BRV) dynamics to different types of cognitive processes (Lenskiy and Paprocki, 2016, unpublished; Gebrehiwot et al., 2017). BRV is a series constructed from stacked-up intervals between eye blinks, which is comparable to the well-established measure of Heart Rate Variability. However, to the best of our knowledge, the question of whether one can predict intelligence from BRV has not been extensively investigated.

In this study, we extracted eye blinks from frontal electrodes of the electroencephalograph (EEG) of subjects while resting and while taking an IQ test. The intervals between peaks of consecutive blinks were stacked up into a series, referred as BRV. We employed the multifractal detrended fluctuation analysis (MFDFA) of each of the BRV series to measure the average rate of change in the variance of inter-blink intervals. We refer to this measure as the α scale exponent. We tested two hypotheses in this study. First, we tested the null hypothesis that BRV while at rest was equal in its α exponents to BRV while solving IQ-test problems. We reasoned that if the hypothesis was rejected, we could conclude that the dynamics of the inter-blink interval were influenced by the mental workload associated with solving IQ problems. It has been shown that BRV dynamics change under mental workload and the rate of change depends on the task, as different types of tasks engage different cognitive processes. For example, BRV dynamics while reading text are smaller compared to resting, whereas they are larger during memory tests (Lenskiy and Paprocki, unpublished).

## 2. Methods

### 2.1. Ethics statement

The procedure was explained to the subjects before the experiment, but the main purpose of the study was revealed only after the experiment, so that the subjects performed without knowing the purpose, which could subconsciously influence their eye blinks. The experiment caused no harm to, nor had other negative consequences on the subjects. All the subjects provided written consent to participate in the experiment. The study was approved with decision number 17101204 by Institutional Review Board affiliated with the Korea University of Technology and Education.

### 2.2. Experimental procedure

The experiment was conducted in a room that eliminated unexpected changes of light, noise, and temperature. We took into account the readiness of the subjects by ensuring they were capable of performing the task (they were rested, not hungry or stressed, and were fully aware of the procedure) and motivated (however, no incentives were offered). Subjects were asked to inform the investigator about their stress levels and fatigue. If necessary, the experiment was postponed. We avoided collecting data during exam periods. The subjects were not under the influence of caffeine or nicotine.

**Figure 1:**
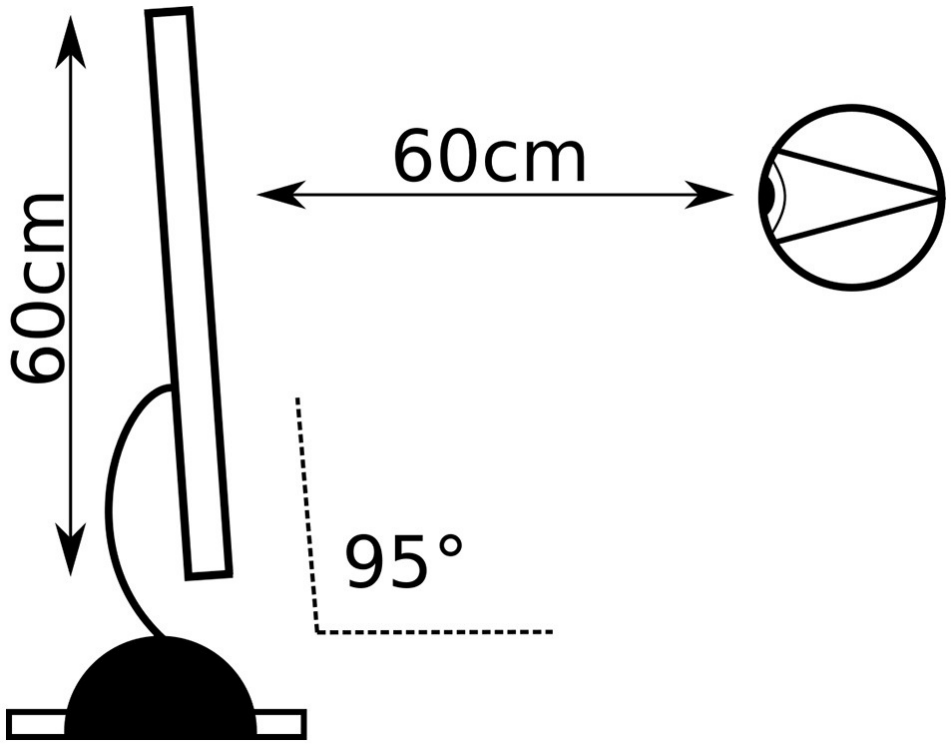
Experimental setup: distance from the screen and visual angle.

**Figure 2:**
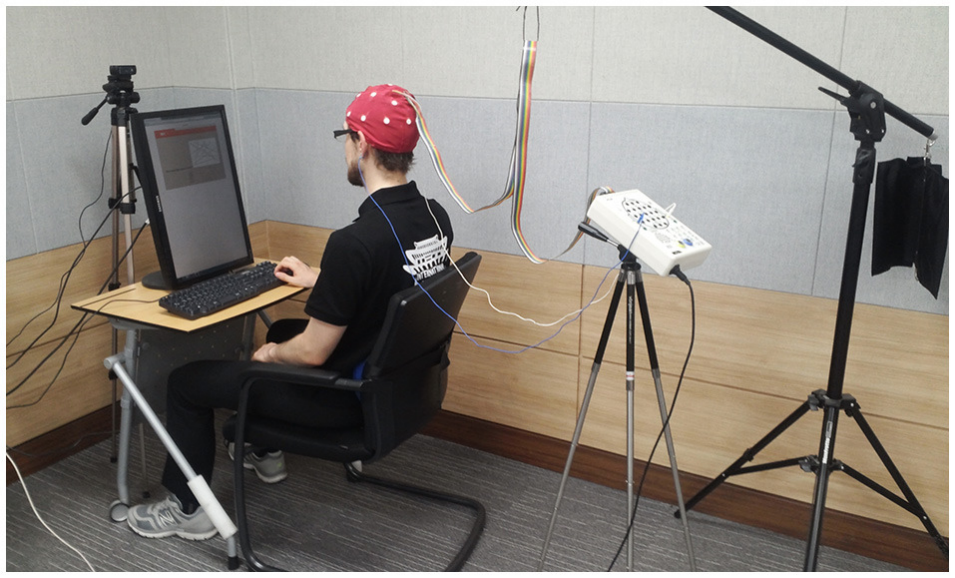
Experimental setup: photograph of actual setup (published by courtesy and consent of the participant).

The subjects were tested individually using a computer. The experiment setup is shown in Figures 1, 2. The experiment consisted of 5 parts: (A) a 5-min resting session, (B) a 10-min IQ-test session, (C) another 5-min resting session, (D) reading a passage of text, and (E) a memory test about the text that was read. In the current work, we focused only on sessions (A) the rest period, and (B) the IQ-test. Electroencephalography (EEG) was used to record the electrical activity of the participants' brains. Next, the eye blinks were extracted from the recorded EEG signals and stacked up into BRV series.

**Figure 3:**
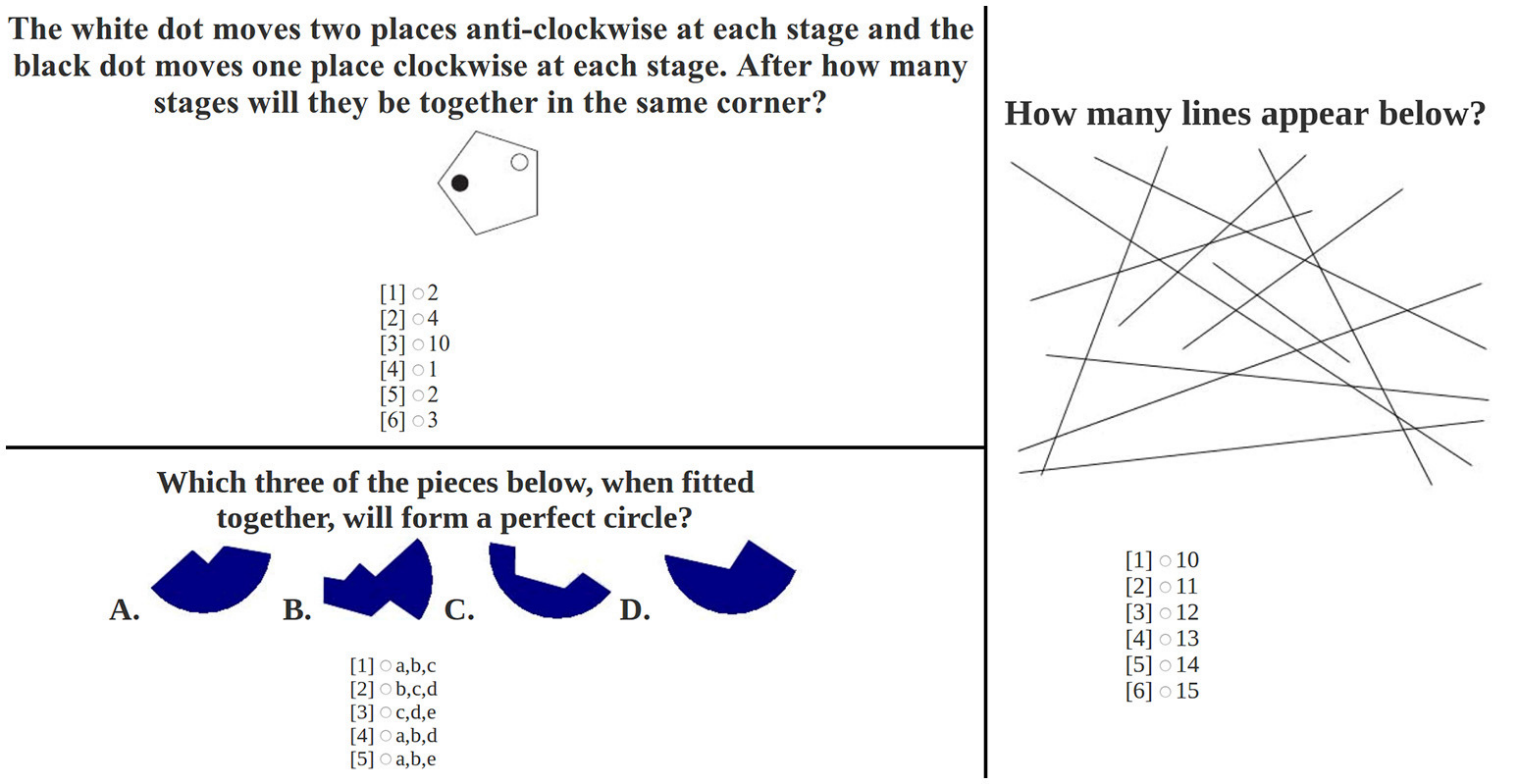
Sample questions from the IQ test.

The IQ test consisted of 13 chosen questions written in English (Figure 3). Because our subjects were not native English speakers, we focused on logical (5 questions), spatial (3 questions), and visual problems (5 questions), and omitted language problems. One point was awarded for each of the 13 problems that was solved correctly. Points were not awarded if a question was not answered within a given amount of time or the answer was incorrect.

In order to minimize head and eye movements, the text was displayed on a single screen in a narrow column. Since EEG is prone to noise due to head and body movements, subjects were asked to sit comfortably and as still as possible, with minimal head movements. The maximum number of answers to each question was limited to 6. The participants answered by pressing buttons from 1 to 6 on the keypad of a keyboard. In order to minimize ambient electrical noise, the EEG amplifier was located approximately two meters away from the computer, and mobile phones and other electronic devices were removed from the room. Video was captured by a webcam; video recordings were used to confirm that subjects did not fall asleep or keep their eyes closed. EEG signals were captured simultaneously. Extraction of eye blinks from EEG is a common problem (e.g., Hoffmann and Falkenstein, 2008; Sovierzoski et al., 2008). The choice of EEG over electro-oculography was motivated by the possibility of analyzing EEG signals in upcoming experiments to provide deeper explanations of the observed phenomena.

### 2.3. EEG

The Mitsar-EEG 201 amplifier and accompanying WinEEG software were used to record EEG. The 19 electrodes were placed according to the “10–20 system.” EEG was recorded with a sampling rate of 250 Hz. We used a mono-polar montage. In the current study, the Fp1 and Fp2 electrodes were our primary interest. They produce a strong electromyographic signal since they are the closest electrodes to the eyes. The recordings were imported into Matlab as CSV files for processing. The data used to conduct this study are available online (Paprocki, 2017).

### 2.4. Data preparation

We collected recordings from twenty-seven subjects (25 males, 2 females), with an average age of 28 ± 7.5 years. The raw data were manually cleaned of artifacts. An algorithm used to extract eye blinks was taken from the EEG based on previous work (Paprocki et al., 2016). Blinks detected by the algorithm were checked manually and confirmed with video recordings. The time intervals between consecutive blinks were stacked up into a series that constituted BRV. One subject who had only a few blinks, which resulted in a short BRV series, had to be excluded. In addition, video recordings showed that two of the remaining 26 subjects were falling asleep during the rest sessions. Therefore, they had to be excluded, as well. Consequently, data from 24 of the 27 subjects remained for further analysis.

### 2.5. Analysis

Physiological systems can be characterized by a power spectra *S*(*f*) with a scaling property $S\left(af\right)\propto \frac{1}{f^{\gamma }}$, where the parameter γ is called the spectral exponent. Many physiological processes exhibit fractal or multifractal properties. Fractal properties of heart rate variability are known to vary with age and various heart conditions (Ivanov et al., 1999). In order to measure fractality of BRV in our work, we employed the α exponent of the MFDFA algorithm. The exponent α is linearly related to the spectral exponent γ via $\alpha =\frac{\gamma +1}{2}$, for γ > − 1. Processes with α = 0.5, 1, and 1.5 describe white, pink, and brown noises, respectively, and are generally called color noise for various values of α. Color noise occurs in many physical, economic, and biological systems, including neural activity and DNA sequences (Bryce and Sprague, 2012). In the present study, the α exponent is a characteristic of BRV dynamics.

An important parameter of DFA is the range of intervals into which the process under investigation is divided. We set the intervals' lengths to: {8, 10, 12, 14, 16, 20, 22, 26, 32, 38, 46, 54, 64} ^2^.

MFDFA is a technique to characterize the multifractal nature of a time series (Kantelhardt et al., 2002). It allows one to estimate the so-called singularity spectrum (MFS) (Kinsner, 2005). The MFDFA is based on detrended fluctuation analysis, which measures the growth of variance in a window of increasing length. Prior to calculating the variance, the time series in the window is detrended by subtracting a polynomial function fitted to the series. This procedure is performed to suppress harmonics whose periods are longer than the window size. Subtraction of the polynomial function is a non-linear procedure that modifies the spectrum in a non-linear fashion. In order to suppress unwanted harmonics outside of the range of the analyzed frequencies, a linear operator is applied instead of polynomial subtraction, as performed in Lenskiy (unpublished). The frequency range is proportional to the window length (scale) *l*.

The estimation algorithm is summarized in the following four steps. First, a sub-band filter with a Gaussian kernel is applied. The spread parameter σ in the Gaussian depends on the scale *l*. Next, the series is divided into *N*_*l*_ overlapping sub-windows of length 2·*l* each. For every sub-window, the variance $\sigma_{k}^{2}(l)$ is estimated. Then, the partition function is calculated as the sum of the variances in each window powered by moment *q*:

$$\begin{array}{l} \chi \left(q,l\right)=\overset{N_{l}}{\underset{k=1}{\sum }}{\left[\sigma_{k}^{2}\left(l\right)\right]}^{q/2} \end{array}$$

The partition function is expected to follow the power law:

$$\begin{array}{l} \chi \left(q,l\right)\propto l^{\tau \left(q\right)} \end{array}$$

where τ(*q*) is related to the multifractal spectrum *f*(α) through a Legendre transform as *f*(α) = *qα* − τ(*q*).

The multifractal spectrum (MFS) *f*(*q*) and Holder exponents α(*q*) are estimated as follows (Yamaguti and Prado, 1995):

$$\begin{array}{l} f(q)=\underset{l\to \;0}{\lim}\frac{\sum_{k=1}^{N_{l}}\mu_{k}(q,l)\text{ln}(\mu_{k}(q,l))}{\text{ln}(l)} \end{array}$$

$$\begin{array}{l} \alpha \left(q\right)=\underset{l\to \;0}{\lim}\frac{\sum_{k=1}^{N_{l}}\mu_{k}\left(q,l\right)\text{ln}\left(\sigma_{k}^{2}\left(l\right)\right)}{\text{ln}\left(l\right)} \end{array}$$

where

$$\begin{array}{l} \mu_{k}\left(q,l\right)=\frac{{\left[\sigma_{k}^{2}\left(l\right)\right]}^{q}}{\chi \left(q,l\right)} \end{array}$$

In our work, we incorporated a single exponent α(*q* = 0) and refer to it as α, which describes the peak of MFS.

### 2.6. Statistical analysis

The normality of BRV was verified using the Shapiro-Wilk test (*p* = 0.05), and one-way ANOVA was used to test the hypotheses.

## 3. Results

The estimated average and standard deviation of the blink rate (BR) was 18.27 ± 10.44 and 19.14 ± 11.1 during the resting and IQ sessions, respectively. The mean of the α exponents was 0.80 ± 0.23 and 0.62 ± 0.16 for the resting and IQ-test sessions, respectively. ANOVA determined that the α distributions of the BR during the resting and IQ-test sessions were significantly different, with *F*_(1, 46)_ = 9.43, *p* = 0.036 and *F*_(1, 46)_ = 12.99, *p* < 0.001. The BRs and α exponents for both sessions (resting and IQ testing), are presented in Table 1 along with the scores of the IQ test.

**Table 1:** The values of BR (blink rate = number of blinks per minute) and α during the resting and IQ-test sessions for all the subjects.

**ID**	**Resting BR**	**Resting α**	**IQ BR**	**IQ α**	**IQ score**
1	19.40	0.77	20.50	0.55	4
2	21.80	0.80	27.40	0.69	5
3	9.20	0.94	4.20	0.16	4
5	23.80	0.50	11.90	0.65	2
6	11.20	0.79	16.80	0.80	2
7	14.80	1.29	9.10	0.69	7
9	12.80	0.61	9.80	0.64	2
10	14.40	0.54	16.50	0.64	4
11	29.80	0.85	41.80	0.58	2
12	11.20	0.73	7.00	0.70	3
13	12.80	1.21	24.10	0.74	6
14	17.40	1.26	34.10	0.60	5
15	10.60	0.59	10.60	0.23	3
16	52.20	0.57	36.70	0.67	3
17	38.60	0.90	16.90	0.59	2
18	10.80	0.89	13.80	0.82	2
19	8.80	0.43	10.20	0.78	4
20	15.00	0.64	12.90	0.52	5
21	19.20	1.01	45.40	0.78	4
22	15.60	0.63	12.50	0.53	6
23	31.00	0.85	18.40	0.61	6
25	10.60	0.88	11.40	0.81	8
26	16.00	0.89	21.70	0.60	6
27	11.40	0.64	25.60	0.62	3
〈*n*〉 ± σ	18.27 ± 10.44	0.80 ± 0.23	19.14 ± 11.10	0.62 ± 0.16	−

*The last row shows the means and the standard deviations*.

Subjects were divided into two groups based upon their IQ scores. The first group consisted of 9 subjects with scores above the median (= 4), and the remaining 15 subjects formed the second group, with scores below or equal to the median. We refer to them as group *IQ*^+^ and *IQ*^−^, respectively.

We used one way ANOVA to analyze the BR and α exponents of the two groups of subjects during the resting and IQ-test sessions (see Table 2). During the resting session, the BR of the *IQ*^+^ group was 16.14 ± 5.72, while the subjects in the *IQ*^−^ group had an average BR of 21.24 ± 14.65. We tested the hypothesis *H*_0_ (i.e., the null hypothesis) that the BR is not an indicator of cognitive performance; the ANOVA results for the resting and IQ sessions were *F*_(1, 22)_ = 0.139, *p* = 0.713 and *F*_(1, 22)_ = 0.001, *p* = 0.981, which was insufficient evidence for rejecting *H*_0_. During the resting session, the exponent α for the *IQ*^+^ group was 0.94 ± 0.25, whereas for the *IQ*^−^ group it was 0.72 ± 0.18. Looking at the IQ-test session, we see the BR for the *IQ*^+^ was 9.59 ± 5.49, whereas for the *IQ*^−^, it was 9.54 ± 5.92. During the IQ-test session, the exponent α for the *IQ*^+^ was 0.64 ± 0.10, whereas for the *IQ*^−^ group, it was 0.61 ± 0.19.

**Table 2:** The mean and the standard deviation of the values of BR and α during the resting and IQ-test sessions of groups *IQ*^+^ and *IQ*^−^.

**Group**	**resting BR**	**resting α**	**IQ BR**	**IQ α**
1 *IQ*^+^	17.22 ± 6.01	0.94 ± 0.25	19.07 ± 8.42	0.64 ± 0.10
1 *IQ*^−^	18.89 ± 12.54	0.72 ± 0.18	19.18 ± 12.72	0.61 ± 0.19
*p*(*F*)	0.713 (0.139)	0.019 (6.456)	0.981 (0.001)	0.677 (0.178)

*The last row shows the ANOVA p-values (F statistic) for between-group differences*.

The α exponents during the IQ test did not indicate any difference between the two groups during the IQ-test session [*F*_(1, 22)_ = 0.178, *p* = 0.677], but there was a group difference in the exponents estimated for the resting session *F*_(1, 22)_ = 6.456, *p* = 0.019. Hence, we accepted the hypothesis that the population means of the α exponents of both groups were different during the resting session. Therefore, the scale exponent estimated during rest may indicate the cognitive performance of the subjects.

**Figure 4:**
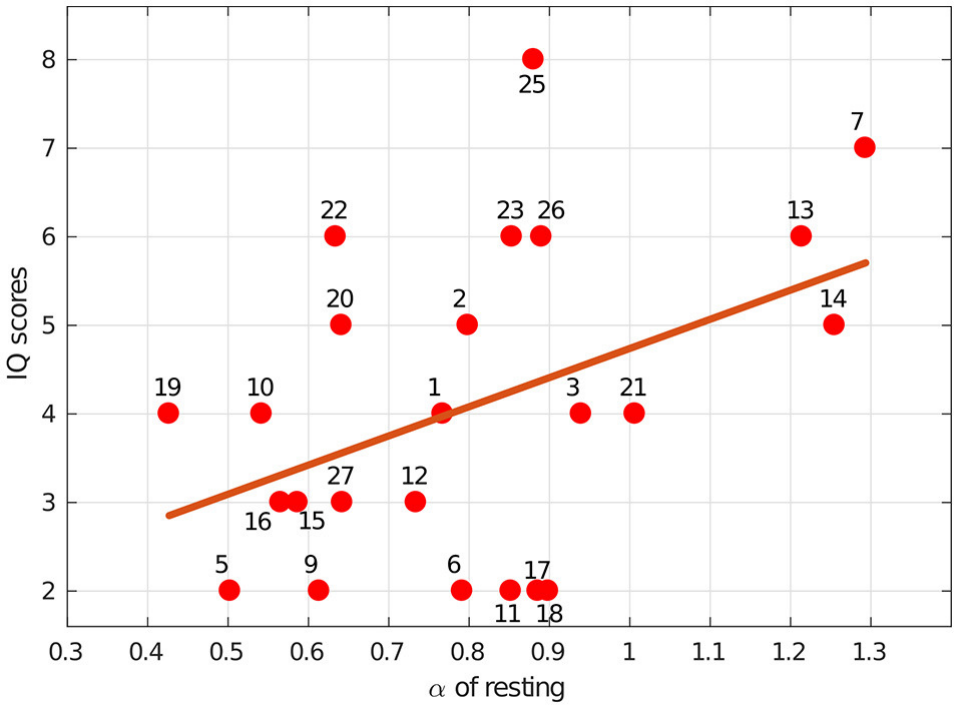
Intelligence, as measured by our IQ-test score, was associated with blink rate variability dynamics. The line indicates the least-squares regression fit [*r*_(22)_ = 0.43, *p* = 0.035, *R*^2^ = 0.185] for IQ scores vs. the α exponents estimated during the resting session. The numbers indicate specific subjects.

**Figure 5:**
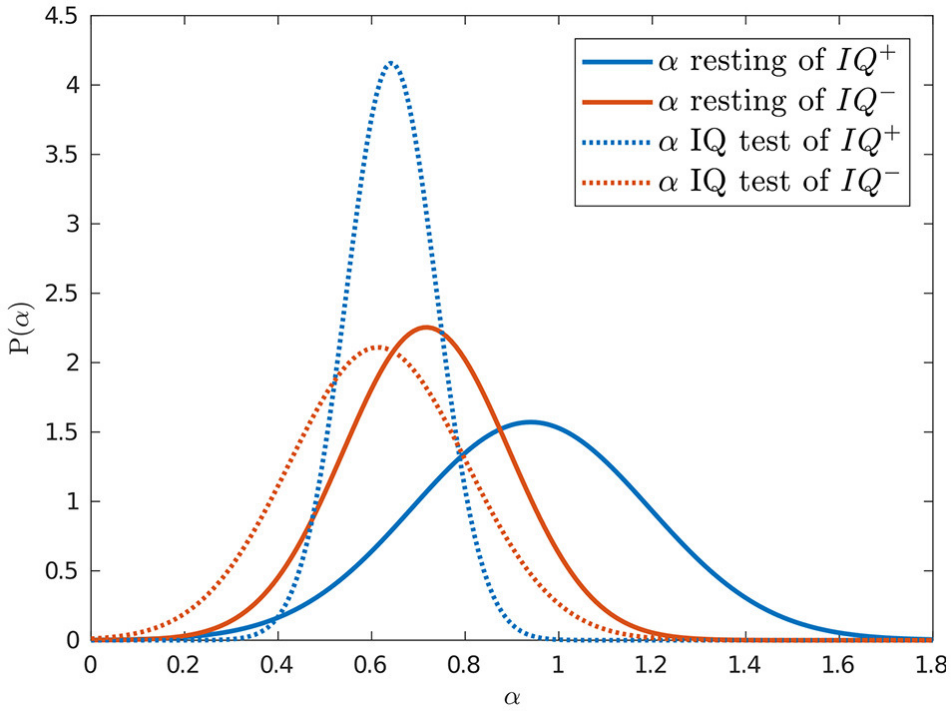
Intelligence was predicted by blink rate variability dynamics while resting. Normal probability density functions were fitted to the estimated α exponents of the resting (solid) and IQ-test (dotted) sessions for the groups with higher (blue) and lower (red) IQ scores.

In summary, we hypothesized that the dynamics of eye blink rate variability are influenced by the mental workload associated with solving IQ problems. This appears to be true, since there was a difference between α while resting and solving IQ-related problems (*p* = 0.004). We also showed that scores on an IQ test were positively correlated with the scale exponent of BRV during rest [with *r*_(22)_ = 0.43, *p* = 0.035, *R*^2^ = 0.185], which can be observed in Figure 4. Additionally, we found that the group with higher IQ scores (*IQ*^+^) had significantly higher α BRV while resting than the group with lower IQ scores did, *IQ*^−^ (*p* = 0.019) (see Figure 5). However, the α of BR did not reveal a difference between those two groups, which suggests BRV might be applied where BR fails.

## 4. Discussion

The present study investigated eye blinks during rest and in the presence of mental workload. Mental workload was manipulated by stimulating cognitive activity in response to answering IQ-test items that focused on mathematical problems, recognition of geometrical patterns, and visual problems; hence, in terms of MI, logical-mathematical and visual-spatial intelligence were tested. Such intelligence requires the ability to stay focused and efficiently use working memory, a system that is driven by dopamine (e.g., Williams and Castner, 2006). By analyzing the α of BRV with respect to IQ scores, we tested whether the dynamics of BRV is a predictive indicator of intelligence.

Convergent thinking (the ability to give a correct answer) brings executive functions into play, which entail a set of cognitive processes that are necessary for the cognitive control of behavior (Diamond, 2013). These functions keep a person focused until a solution is found. It has been shown that functions related to the frontal lobe, including working memory, are responsible for maintaining a high level of focus on a task (Duncan et al., 2000). This phenomenon aligns and binds other cognitive processes and keep individuals focused on a task (Chermahini and Hommel, 2010). On the other hand, it has been shown that dopamine regulates blinking (Jongkees and Colzato, 2016). The basal ganglia, which are interconnected with the cerebral cortex (Bostan et al., 2013) and play a key role in memory, attention, and consciousness, modulate the release of dopamine in the striatum, thereby influencing the eye-blink reflex (Evinger et al., 1993). The basal ganglia also control the input of working memory (WM), and have the capacity to manipulate information in short-term memory and use it to guide action (Baddeley, 1998). It has been proposed that one of the functions of the basal ganglia is to filter what enters into working memory and modulate its focus by modifying dopamine levels (Schroll and Hamker, 2013). This phenomenon has also been used to support eye-blink rate as a measure to track changes in WM during task performance and as a possible measure of striatal dopamine activity (Rac-Lubashevsky et al., 2017). In our research, the phenomena of gating information and exciting basal ganglia circuits on a given task can be observed in the difference (*p* = 0.0036) between the α values during the IQ-test and the resting session. The change in BRV dynamics might suggest a change in dopamine levels, although further research is required to explore this possibility. A conceptually similar phenomenon has been observed by Tsukahara et al. (2016), indicating a relationship between cognitive abilities and pupil size during a passive baseline condition, with *r*_(60)_ = 0.34, *p* < 0.05, *r*_(205)_ = 0.25, *p* < 0.05, and *r*_(60)_ = 0.38, *p* < 0.05, in Caucasian, African-American, and Other subjects, respectively. Their finding that pupil size in passive conditions predicted cognitive abilities is similar to our result that BRV dynamics while resting indicated the subjects' cognitive state, with *r*_(22)_ = 0.43, *p* = 0.035.

The substantia nigra pars compacta, which is part of the basal ganglia, contributes to reward-seeking, addiction, and eye movements. Additionally, there is evidence that this limbic portion of the basal ganglia plays a key role in reward learning. Extracellular dopamine has a substantial influence on limbic-basal ganglia circuits. Among the drugs that work by increasing the efficacy of the dopamine signal, the most addictive drugs are cocaine, amphetamine, and nicotine. Montague et al. (2004) discussed the way the basal ganglia incorporate dopamine, not only for reward and focus, but to maintain task- and goal-relevant information: an “Important component of dopaminergic gating takes place in the basal ganglia, acting selectively on recurrent pathways that run from the PFC through the basal ganglia and back to the PFC.” This mechanism allows for selectively updating goal representations within the prefrontal cortex (PFC). Furthermore, the association cortex of the frontal lobe has been found to be related to working memory (Sasaki et al., 1994). Moreover, the PFC, which is in the inferior portion of the frontal lobe, functionally influences eye-blinking (Weiss and Disterhoft, 2011). The neural network activity of the PFC has been shown to change behavior during working memory under the influence of dopamine (Surmeier, 2007). In fact, dopamine is secreted in the PFC during higher-order executive functions, such as learning, memorizing, and recall of memories (Puig et al., 2014). This points to the importance of dopaminergic mechanisms for cognitive performance.

Apart from being crucial for various higher-order functions, dopamine is linked to eye-blinking, specifically, the BR, which is a marker of dopaminergic functioning in the striatum, one of the basal ganglia's nuclei (Shukla, 1985). A clear connection between blinking and dopamine was observed in an experiment with recreational cocaine users. Correlation analyses showed that recreational cocaine users had a significantly reduced BR compared to a cocaine-free group (Colzato et al., 2008). To summarize, there is a clear connection between cognitive performance and dopamine, which influences eye-blink phenomena.

In addition to dopamine, GABA is a neurotransmitter that is known to be related to eye blinks and correlated with level of intelligence (Colzato et al., 2008). Cook et al. (2016) demonstrated a strong positive correlation between visual intelligence and cortical GABA concentrations (*r* = 0.83, *p* = 0.005). Subjects with high levels of GABA in the primary visual cortex performed better on a matrix reasoning IQ sub-test. It is also known that blinking behavior is under perceptual and cognitive control (Orchard and Stern, 1991) and that it is associated with the suppression of visual cortex activity (Bristow et al., 2005). Since it has been shown that the primary visual cortex is modulated during eye blinks (Williams et al., 2008), we suspect that blinking behavior is linked to GABA accumulation in the visual cortex; thus, it may affect eye-blink characteristics as well. However, that requires further investigation.

## 5. Limitations

The limitations of this study point to possible directions for further research. First, questions were chosen from pattern recognition, visual-spatial, and logical-mathematical domains. The number of questions needs to be increased to analyze those domains thoroughly, and those domains probably should be divided into separate sessions. An updated IQ-test and various cognitive tests should also be used in future research. Possible items could be chosen from tests like Raven's Progressive Matrices (Raven, 1936), the Stanford–Binet (Becker, 2003), the Multidimensional Aptitude Battery II (Krieshok and Harrington, 1985), and other IQ tests.

Second, the results of IQ-tests are affected by both intelligence and test motivation. It has been shown that incentives motivate subjects and increase their scores by 0.96 SD and 0.26 SD for groups with below-average and above-average IQs, respectively (Duckworth et al., 2011). However, with no reward, the latter group performs closer to its maximum potential than the former group. The fact that subjects' motivation on tests can complicate the interpretation of IQ results should be taken into consideration.

Third, as we discussed above, BR is a marker of current dopamine level, and it is known that dopamine is a hormone associated with happiness (Sharot et al., 2009). In particular, Akbari Chermahini and Hommel (2012) demonstrated that positive mood leads to an increase in BR and cognitive flexibility, i.e., the ability to adapt cognitive processing strategies to solve new tasks. Hence, the subjects' mood has to be taken into account in future experiments. Finally, although the sample size we used in this experiment is consistent with that used in this general area of research, a larger sample size would have provided greater statistical power to our analyses. Thus, we recommend that future studies use larger sample sizes to be better able to interpret the results.

## 6. Conclusion

This study demonstrated a statistically significant correlation of the α exponents estimated for BRV during rest and IQ-test scores. Since eye-blinks are connected to higher cognitive processes, we hypothesize that BRV dynamics can be used as a marker of dopa- and gabaminergic functioning. Additionally, the α of the BRV of the *IQ*^+^ group was significantly higher compared to the *IQ*^−^ group while resting. The findings confirm the already known phenomenon of a relationship between cognitive abilities and pupil size during a passive baseline condition. The finding that pupil size in passive conditions predicts cognitive abilities is similar to our finding that BRV dynamics during rest indicates a subject's cognitive state. This result suggests the possibility of comparing cognitive performance among subjects without having them perform any tasks. The significance of our findings is that BRV can be employed in a broad spectrum of cognitive experiments. It can be helpful in studies to understand the role of fluid intelligence as well as studies on the resting-state brain. BRV can also be useful to assess mental workload, e.g., as part of a combined measure (Ryu and Myung, 2005). This study provides a foundation for further studies assessing cognitive functioning based on the measurement of BRV. To summarize, the findings of this study revealed that the scale exponent α of blink rate variability dynamics can be used as an indicator of cognitive performance while the brain is not occupied by a task. The nature of this link remains to be elucidated by further research.

## Author contributions

RP and AL designed the study and wrote the manuscript. AL developed the methodology. RP collected the data and performed the analysis.

### Conflict of interest statement

The authors declare that the research was conducted in the absence of any commercial or financial relationships that could be construed as a potential conflict of interest.

## Footnotes

^1^The search was conducted on 2017-06-01.

^2^according to formula *l* = 2· *round*(2^*n*^), *where n is* [2:0.25:5].
